# Metabolomic and Physiological Analyses Reveal the Effects of Different Storage Conditions on *Sinojackia xylocarpa* Hu Seeds

**DOI:** 10.3390/metabo14090503

**Published:** 2024-09-18

**Authors:** Hao Cai, Yongbao Shen

**Affiliations:** Collaborative Innovation Centre of Sustainable Forestry in Southern China, College of Forestry, Nanjing Forestry University, Nanjing 210037, China

**Keywords:** *Sinojackia xylocarpa* Hu, seed storage, physiology, metabolomics

## Abstract

Backgrounds: *Sinojackia xylocarpa* Hu is a deciduous tree in the *Styracaceae* family, and it is classified as a Class II endangered plant in China. Seed storage technology is an effective means of conserving germplasm resources, but the effects of different storage conditions on the quality and associated metabolism of *S. xylocarpa* seeds remain unclear. This study analyzed the physiological and metabolic characteristics of *S. xylocarpa* seeds under four storage conditions. Results: Our findings demonstrate that reducing seed moisture content and storage temperature effectively prolongs storage life. Seeds stored under that condition exhibited higher internal nutrient levels, lower endogenous abscisic acid (ABA) hormone levels, and elevated gibberellic acid (GA_3_) levels. Additionally, 335 metabolites were identified under four different storage conditions. The analysis indicates that *S. xylocarpa* seeds extend seed longevity and maintain cellular structural stability mainly by regulating the changes in metabolites related to lipid, amino acid, carbohydrate, and carotenoid metabolic pathways under the storage conditions of a low temperature and low seed moisture. Conclusions: These findings provide new insights at the physiological and metabolic levels into how these storage conditions extend seed longevity while also offering effective storage strategies for preserving the germplasm resources of *S. xylocarpa*.

## 1. Introduction

*Sinojackia xylocarpa* Hu is a deciduous tree species belonging to the *Styracaceae* family. Its white, fragrant flowers and weight-like fruit make it highly ornamental [1,2]. However, due to continuous habitat destruction, the population of this wild plant has severely declined. Additionally, the mechanical constraint of the seed coat makes it difficult for the seeds to germinate naturally, which accelerates the endangerment of this species [3]. Consequently, *S. xylocarpa* has been classified as a Class II endangered plant in China [4]. Effective conservation measures are urgently needed to prevent its extinction in the wild. Seed storage technology is an effective method to preserve the plant’s valuable germplasm resources. Currently, China has successfully preserved the seeds of the endangered tree species *Calocedrus macrolepis*. After being stored in a seed bank for 5 years, these seeds still maintain high viability [5].

Seed storage involves using appropriate storage equipment and advanced scientific techniques to reduce seed respiration activity, delay seed aging as much as possible, and effectively maintain seed viability [6]. The effectiveness of seed storage largely depends on the genetic characteristics of the seeds themselves, as well as on seed moisture content and storage temperatures. Studies have shown that reducing the seed moisture content of seeds such as onion (*Allium cepa*), rice (*Oryza sativa*), and *Moringa oleifera* and storing them in low-temperature environments can effectively maintain seed viability over the long term [7,8,9]. However, not all seeds should have their moisture content and storage temperature reduced as much as possible. For instance, seeds of *Ginkgo biloba* and *Quercus acutissima* are sensitive to dehydration [10,11]. Once dehydrated, these seeds cannot survive, and ice crystals easily form in low-temperature environments, ultimately leading to seed death [12]. Therefore, storage temperature and seed moisture content significantly impact the storage effectiveness of seeds, and the optimal storage temperature and moisture content vary among different species.

The current research on seed storage mainly focuses on physiological, biochemical, and cellular structural changes [9,13], while studies on seed metabolites during storage remain relatively scarce. Metabolomics is an emerging and highly promising discipline in the field of plant science [14]. Metabolites are the end products of cellular metabolic processes, and their levels can be regarded as the ultimate response of an organism to genetic or environmental changes. Various metabolic changes occur during the normal storage of seeds, such as changes in stored nutrients and carbohydrates, which provide energy for seed respiration during storage. Therefore, understanding the metabolic changes during seed storage can provide deeper insights into the potential mechanisms of seed aging. Lv used liquid chromatography–mass spectrometry (LC-MS) to study the potential marker substances of Chinese cabbage (*Brassica pekinensis*) seeds stored for 13 years, and they successfully identified multiple key metabolites involved in seed deterioration processes, such as mold-induced compounds, which were mainly enriched in the flavonoid biosynthesis process [15]. Therefore, identifying changes in seed metabolites under different storage conditions is crucial for optimizing seed storage strategies and extending seed longevity.

As the preservers and transmitters of plant genetic information, seeds are ideal materials for the long-term preservation of plant germplasm resources. Seed storage is the most common, reliable, and practical method for preserving plant germplasm resources. Therefore, comprehensively and accurately understanding the physiological, biochemical, and metabolomic changes during the storage process is crucial for formulating seed storage strategies for endangered species. The findings of this study will provide theoretical support for the scientific storage of seeds of the endangered species *S. xylocarpa* and offer important references for the conservation of other native endangered tree species.

## 2. Materials and Methods

### 2.1. Experimental Materials

The experimental materials were collected in October 2022 from the Nanjing Forestry University Xiasu Experimental Forest, Xiasu Town, Jurong City, Jiangsu Province (119°13′30″ E, 32°7′33″ N). Each fruit contained a single seed enclosed within a thick, woody pericarp. The seeds were dark brown and cylindrical. The endosperm was fleshy, and the embryo was linear and elongated. The fruits had an average longitudinal diameter of 24.4 ± 2.6 mm, a transverse diameter of 13.3 ± 2.1 mm, and an initial seed moisture content of 40.3%. Fresh *S. xylocarpa* seeds extracted from fruits were naturally dried to moisture contents of 10.1% and 7.3% at room temperature (10~25 °C) and then stored in plastic bags.

### 2.2. Experimental Methods

Upon reaching the target moisture content, the seeds were subjected to four storage conditions: H-St (seed moisture content of 10.1%, storage temperature of 4 ± 2 °C), H-Lt (seed moisture content of 10.1%, storage temperature of −18 ± 2 °C), L-St (seed moisture content of 7.3%, storage temperature of 4 ± 2 °C), and L-Lt (seed moisture content of 7.3%, storage temperature of −18 ± 2 °C). The storage duration was from December 2022 to November 2023. Samples were collected at 60, 120, 180, 240, and 300 d of storage. For each sampling, 50 seeds were taken, processed by removing the seed coat, homogenizing the kernels, and stored at −80 °C until metabolomics and biochemical analyses were conducted.

### 2.3. Seed Viability Assay

Seed viability was determined using the tetrazolium staining method (TTC). Three sets of 50 seeds each were randomly selected (quartering method), soaked at 25 °C for 24 h, dehulled, immersed in a 0.5% TTC solution, and stained in the dark at 37 °C for 10 h. Seeds with more than 75% of their embryo area stained and showing root staining were considered viable [16].

### 2.4. Physiological and Biochemical Assays

Total soluble protein, soluble sugar, starch, and fat contents were quantified at 60, 120, 180, 240, and 300 days of seed storage. Soluble protein was measured using the Bradford method [17], with bovine serum albumin as the standard. Soluble sugars and starch were quantified using the anthrone colorimetric method [18,19], and fat content was determined through Soxhlet extraction [20].

Endogenous hormone levels, including abscisic acid (ABA), indole-3-acetic acid (IAA), gibberellic acid (GA₃), and zeatin riboside (ZR), were extracted, purified, and quantified using enzyme-linked immunosorbent assays (ELISA), following the protocols described by Zhao et al. [21] and Wang et al. [22]. The ELISA kits were provided by the Laboratory of Plant Endogenous Hormones, China Agricultural University. To extract the hormones, 10 mL of cold 80% (*v*/*v*) methanol extraction solution containing 1 mmol·L^−1^ of butylated hydroxytoluene (BHT) as an antioxidant was used. The extracts were incubated at 4 °C for 4 h and centrifuged at 1000 rpm for 15 min at 4 °C. The resulting supernatant was filtered through a Sep-Pak C18 column (Waters Corp., Milford, MA, USA), and it was washed with 10 mL of 100% (*v*/*v*) and 5 mL of 80% (*v*/*v*) methanol, respectively. Hormonal components were then eluted with 10 mL of 100% (*v*/*v*) methanol and 10 mL of diethyl ether. The eluted fractions were dried under nitrogen gas and re-dissolved in 2 mL of phosphate-buffered saline (PBS) containing 0.1% (*v*/*v*) Tween 20 and 0.1% (*w*/*v*) gelatin (pH 7.5) for ELISA analysis. Standards and samples (50 μL each) were loaded in duplicate into a 96-well ELISA plate, and 50 μL of specific primary antibody was added to initiate competitive binding. After incubation, the wells were washed, and 100 μL of enzyme-labeled secondary antibody was added. The reaction was developed using orthophenylenediamine (OPD) as the chromogen, and absorbance was measured at 450 nm using an ELISA reader. Hormone concentrations were expressed as ng·g^−1^ FW. Each experiment was performed in triplicate, and the results were reported as means ± standard errors.

### 2.5. Transmission Electron Microscopy (TEM)

To better understand the effects of storage conditions on embryo cell morphology, embryos from each storage condition were selected for TEM observation in order to observe relevant microstructural changes [23]. Initially, embryos from both non-stored seeds and seeds stored under different conditions for 300 d were fixed in 2% glutaraldehyde in 0.1 M PBS buffer for 24 h. Subsequently, samples were rinsed with PBS and post-fixed in 1% OsO4 for 2 h before TEM analysis. Dehydration involved sequential immersion in graded ethanol concentrations (30%, 50%, 70%, and 90%) for 15 min each, followed by graded acetone concentrations (90% and 95%) for 15 min each, and then in a series of resin mixtures (1:1 and 3:1) for 1 to 3 h. Samples were embedded in 100% Spurr resin and sectioned into 70 nm ultra-thin slices using an ultramicrotome. Sections were stained with 1% toluidine blue (TBO) and observed using a transmission electron microscope (Hitachi H-7650, Hitachi, Japan) to examine embryo cell structures.

### 2.6. Metabolite Profiling

Referencing Lv’s experimental methods [15], metabolite analysis was conducted as follows: First, embryos of *S. xylocarpa* were subjected to vacuum freeze-drying using a Scientz−100F freeze dryer. Samples were then ground into powder using an MM 400 grinder (Retsch). Fifty mg of sample powder was weighed and mixed with 1200 μL of a −20 °C pre-cooled 70% methanol aqueous solution containing internal standards. The mixture was vortexed for 30 s every 30 min, which was repeated 6 times. After centrifugation (12,000 rpm, 3 min), the supernatant was collected, filtered through a 0.22-μm-pore-size membrane filter, and stored in injection vials for liquid chromatography–tandem mass spectrometry (LC-MS/MS) analysis. All samples were analyzed using an LC-MS system, following machine instructions.

The LC-MS/MS analysis conditions were as follows: The chromatographic column used was Waters Acquity UPLC HSS T3 (1.8 µm, 2.1 × 100 mm). Mobile phase A consisted of 0.1% formic acid in water, and mobile phase B consisted of 0.1% formic acid in acetonitrile. The gradient elution program started at 95% A, 5% B, was programmed to 35% A, 65% B within 5 min and then to 1% A, 99% B within 1 min, and was held for 90 s, followed by a 6-s adjustment back to 95% A, 5% B, and a 144-s hold. Data acquisition was performed using the Analyst TF 1.7.1 software (Sciex, Concord, ON, Canada) in IDA mode.

The ESI source parameters were set as follows: A sheath gas flow rate at 50 psi, an ion source gas 1 at 60 psi, and curtain gas at 35 psi. The temperature was set to 550 °C (TEM). The declustering potentials (DPs) were 80 V in the positive and negative ion modes. The ion spray voltages floating (ISVF) were 5.5 kV in the positive mode and 4.5 kV in the negative mode. Collision energies were set to 30 V for both the positive and negative ion modes.

### 2.7. Data Processing and Statistical Analysis

Statistical analyses were performed using SPSS 22.0 and Microsoft Office Excel 2019. A one-way analysis of variance (ANOVA) and Duncan’s multiple range test were used to determine significant differences among the treatments, and significance was set to *p* < 0.05. Orthogonal partial least squares discriminant analysis (OPLS-DA) was used for metabolite differences between storage conditions, with variable importance in projection (VIP) selection criteria (VIP > 1, *p* < 0.05, fold change (FC) ≥ 1.5 or ≤−1.5). Principal component analysis (PCA) was conducted using the R package prcomp (3.5.0). Pathway enrichment analysis was performed using Kyoto encyclopedia of genes and genomes (KEGG) databases, applying the hypergeometric distribution test.

## 3. Results

### 3.1. Impacts of Different Storage Conditions on the Seed Quality of S. xylocarpa Seed

**Figure 1:**
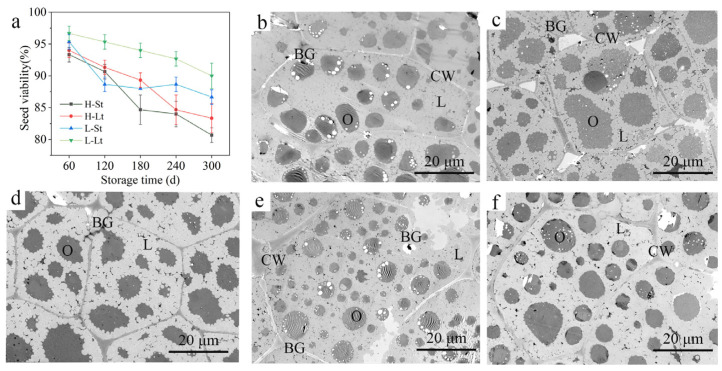
Changes in seed quality during seed storage of *S. xylocarpa*: (**a**) seed viability, (**b**–**f**) seed ultrastructure. BG, highly electron-dense substances; L, lipid droplet; CW, cell wall; O, organelle.

The viability of *S. xylocarpa* seeds under different storage conditions showed a fluctuating downward trend during storage (Figure 1a). By the end of the storage period, the viability of the seeds across all storage conditions could generally be maintained above 80%. Particularly, seeds stored under low-seed-moisture-content and low-storage-temperature (L-Lt) conditions consistently exhibited significantly higher viability throughout the storage period. Conversely, seeds subjected to high seed moisture content and temperature (H-St) conditions showed the lowest viability. By the end of the storage period, seeds in the H-St storage condition experienced the largest decline, decreasing by 12.7%. The viability of *S. xylocarpa* seeds stored under H-Lt and L-St storage conditions decreased by 10.7% and 10.0%, respectively. Fresh *S. xylocarpa* seeds exhibited intact cell membrane systems, distinct organelles, and a dense arrangement of lipid droplets with sparse highly electron-dense materials in the cytoplasm (Figure 1b). After storage 300 d, seeds in the H-St condition showed significant separation between plasma membranes and cell walls; the organelles were indistinct, clustered together and forming large, conspicuous aggregates within cells. The lipid droplets degraded significantly, containing numerous irregularly shaped and variably sized, highly electron-dense materials (Figure 1c). In the H-Lt storage condition, the organelles appeared irregularly shaped, with highly electron-dense materials distributed among the lipid droplets (Figure 1d). The L-St condition exhibited blurred cell membrane structures and a large distribution of organelles of varying sizes, with noticeable highly electron-dense materials observed within the cell cavity (Figure 1e). In contrast, the L-Lt storage condition maintained a clear outer cell wall structure, with only small, sparse, highly electron-dense materials observed between lipid droplets (Figure 1f). Therefore, compared to fresh *S. xylocarpa* seeds, the differences in ultrastructure were minimal in the L-Lt conditions. Overall, L-Lt conditions involving alow seed moisture content and alow storage temperature are most conducive to maintaining seed viability over extended periods. This finding provides crucial scientific insights for optimizing seed storage strategies.

### 3.2. Impacts of Different Storage Conditions on Nutritional Components of S. xylocarpa Seeds

**Figure 2:**
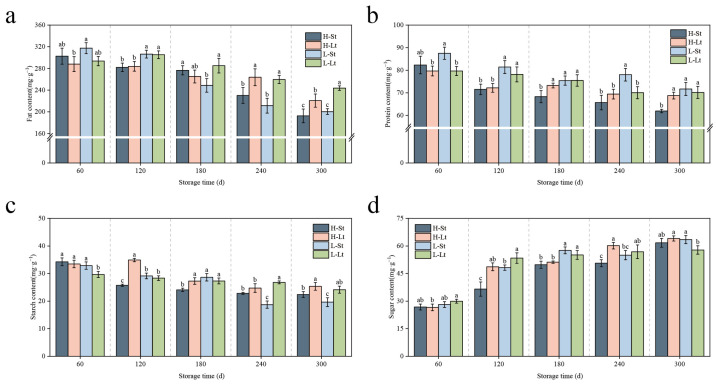
Changes in nutrients during seed storage of *S. xylocarpa*: (**a**) fat content (mg·g^−1^), (**b**) protein content (mg·g^−1^), (**c**) starch content (mg·g^−1^), (**d**) sugar content (mg·g^−1^). All values are expressed as means ± SDs (n = 3). Different lowercase letters in the same column indicate that the difference between values reached a significant level (*p* < 0.05).

During seed storage, significant differences (*p* < 0.05) were observed in the fat, soluble protein, soluble sugar, and soluble starch contents of *S. xylocarpa* seeds under different storage conditions (Figure 2). Throughout the storage period, soluble sugar content exhibited a decreasing trend across all four storage conditions (Figure 2a). At 60 d, there was no significant difference in sugar content across the different storage conditions. By 300 d, the sugar content reached its peak, with the L-Lt condition showing the highest sugar content and H-St condition showing the lowest. Compared to storage for 60 d, the soluble sugar content increased by 130%, 141%, 125%, and 93% under the respective conditions. At 60 d, there was some variability in the starch content among the different conditions (Figure 2b). Over time, the starch content generally decreased under all conditions. The L-Lt and H-Lt conditions maintained relatively higher starch content compared to L-St and H-St at 300 d. This suggests that lower temperature conditions are more effective in preserving starch content. At 60 d, the protein content showed significant differences among storage conditions, with L-Lt having the highest and H-St the lowest (Figure 2c). The soluble protein content decreased over the entire storage period, with the H-St storage condition significantly lower than other storage conditions by the end of storage (*p* < 0.05). The trends in soluble protein content mirrored those of fat content, both showing a decline (Figure 2d). By 300 d, the fat content under L-Lt condition was the highest, while H-St had the lowest. An analysis of the figures suggests that lower moisture content and lower temperature conditions (L-Lt) are the most effective in preserving the nutritional content of *S. xylocarpa* seeds.

### 3.3. Effects of Different Storage Conditions on Endogenous Hormones in S. xylocarpa Seeds

**Figure 3:**
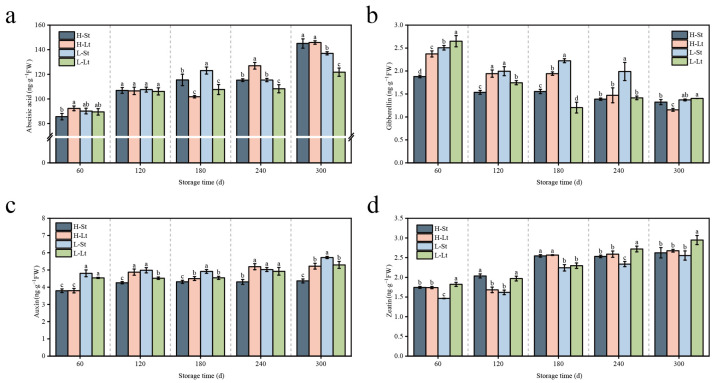
Changes in endogenous hormone during seed storage of *S. xylocarp*: (**a**) abscisic acid content (ng g^−1^FW), (**b**) gibberellin content (ng g^−1^FW), (**c**) auxin content (ng g^−1^FW), (**d**) zeatin content (ng g^−1^FW). All values are expressed as means ± SDs (n = 3). Different lowercase letters in the same column indicate that the difference between values reached a significant level (*p* < 0.05).

During storage, the contents of ABA, ZR, and iIAA in *S. xylocarpa* seeds showed an increasing trend, while GA_3_ exhibited a decreasing trend (Figure 3). At 60 days, there was some variability in gibberellin content among the different conditions (Figure 3a). By 300 d, the ABA content reached its peak, with the H-Lt condition showing the highest ABA content and the L-St condition showing the lowest (*p* < 0.05). Over time, the ABA content increased by 70%, 58%, 52%, and 36% under the H-St, H-Lt, L-Lt, and L-St storage conditions, respectively. At 60 d, there was also some variability in the GA_3_ content among the different conditions (Figure 3b). By 300 d, the GA_3_ content under L-St and L-Lt conditions was relatively higher compared to H-Lt and H-St. Overall, the IAA content showed minimal variation, with the highest content observed in the L-St storage condition by the end of storage, followed by the L-Lt and H-Lt storage conditions, and the lowest in the H-St condition (Figure 3c). At 60 d, the ZR content was relatively low across all storage conditions (Figure 3d). By 300 d, the ZR content under the L-Lt condition was the highest, while the H-St condition had the lowest. Compared to the initial storage period, the ZR content increased by 50%, 54%, 74%, and 62% under the H-St, H-Lt, L-Lt, and L-St storage conditions, respectively.

### 3.4. Differences in Seed Metabolites and Metabolic Pathways under Different Storage Conditions

**Figure 4:**
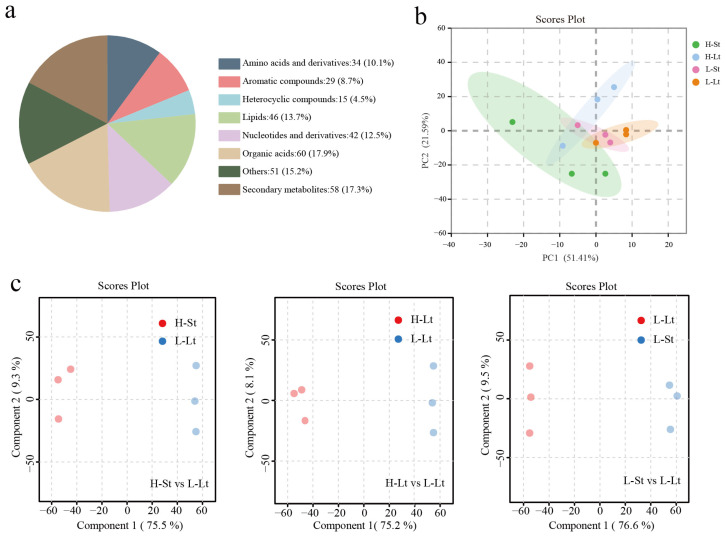
Overview of the detected metabolites in the *S. xylocarp* seeds: (**a**) Kyoto encyclopedia of genes and genomes (KEGG) compound classification of metabolites, (**b**) Principal component analysis (PCA) of metabolites in positive and negative modes, (**c**) Orthogonal partial least squares discriminant analysis (OPLS-DA) score in positive and negative modes.

To comprehensively understand the metabolic differences of *S. xylocarpa* seeds under different storage conditions, we performed untargeted metabolomics analysis using the LC-MS/MS. A total of 4179 intracellular metabolites were detected across all samples, among which 335 metabolites were annotated with KEGG identifiers (Table S1). Furthermore, to show the classification of metabolites more intuitively, we conducted a statistical analysis of the metabolites and analyzed the metabolites. Secondary metabolites, organic acids, and lipids were the top three categories, and each category accounted for more than 10% of the total metabolites (Figure 4a). The PCA indicated a clear differentiation among the storage conditions (Figure 4b). The OPLS-DA was employed to analyze the changes in metabolites of *S. xylocarpa* seeds under different storage conditions (Figure 4c). The results demonstrated significant separation among all conditions, with confidence intervals exceeding 80% for each group. This suggests that the OPLS-DA model was robust, without overfitting, and that it had high reliability and reproducibility for subsequent analyses.

#### 3.4.1. Differential Accumulation Analysis of Metabolites

**Figure 5:**
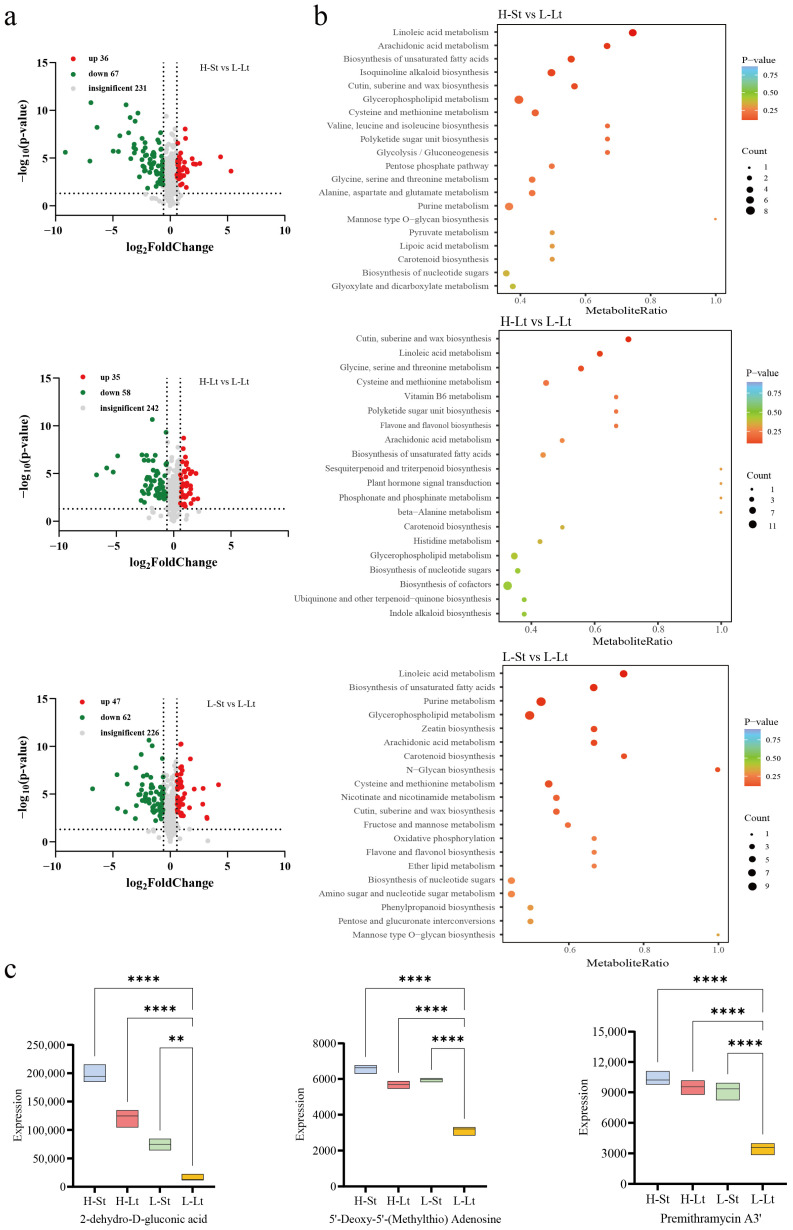
Metabolome analysis of *S. xylocarp* seeds under different storage conditions: (**a**) volcano plots comparing different storage conditions, (**b**) KEGG pathway-enrichment bubble plot of differential accumulated metabolites under different storage conditions of the top 20, (**c**) three representative differentially expressed metabolites. ** represents a significant difference at *p*< 0.01, and **** represents a significant difference at *p* < 0.0001 according to Tukey’s test in a box violin plot.

To expedite the identification of differential metabolites, volcano plots were generated using selection criteria (FC ≥ 1.5 or ≤ −1.5; *p* < 0.05) to pinpoint metabolites of the highest significance (Figure 5a). The volcano plots depict metabolites, with each point representing a specific compound; red indicates upregulation, and blue indicates downregulation. In the comparison between the H-St and L-Lt conditions, 36 compounds showed upregulation, while 67 compounds were downregulated. Comparing different seed moisture levels (H-Lt vs. L-Lt) revealed that 35 compounds were upregulated, and 58 compounds were downregulated. Lastly, comparing different temperatures (L-St vs. L-Lt) indicated that storage temperatures influenced the upregulation of 47 compounds and the downregulation of 62 compounds. The screened differential metabolites were mapped to the KEGG pathway database. A topological analysis was conducted to further filter the enriched pathways, identifying key metabolic pathways more closely associated with the differential metabolites. The results were visually represented using a bubble plot, with each bubble corresponding to a KEGG pathway (Figure 5b). In comparison, between HSt and L-Lt, these metabolites were collectively and significantly enriched in 70 metabolic pathways, including Cutin, suberine and wax biosynthesis, linoleic acid metabolism, glycine, serine, and threonine metabolism, etc. In the comparison between H-Lt and L-Lt, these metabolites were collectively enriched in 72 metabolic pathways, with linoleic acid metabolism, arachidonic acid metabolism, the biosynthesis of unsaturated fatty acids, etc., being significantly enriched. In the comparison between L-St and L-Lt, these metabolites were collectively enriched in 72 metabolic pathways, with linoleic acid metabolism, the biosynthesis of unsaturated fatty acids, purine metabolism, etc., being significantly enriched. Among them, most of the pathways identified were interconnected with lipid, carbohydrate, and amino acid pathways. We focused on the same metabolic pathways under different storage conditions to minimize the impact of different groups. Specifically, the biosynthesis of nucleotide sugars, carotenoid biosynthesis, cysteine and methionine metabolism, glycerophospholipid metabolism, cutin, suberine and wax biosynthesis, the biosynthesis of unsaturated fatty acids, arachidonic acid metabolism, and linoleic acid metabolism are critical pathways influencing the quality of *S. xylocarpa* seeds during storage. Figure 5c shows three representative differential metabolites, namely 2-dehydro-D-gluconic acid, 5′-deoxy-5′-(methyl thio) adenosine, and Premithramycin A3′. Notably, the 2-dehydro-D-gluconic acid expression was the highest in all three comparison groups, at 12.7-fold (H-St vs. L-Lt), 7.8-fold (H-Lt vs. L-Lt), and 4.7-fold (L-St vs. L-Lt) the L-Lt.

**Figure 6:**
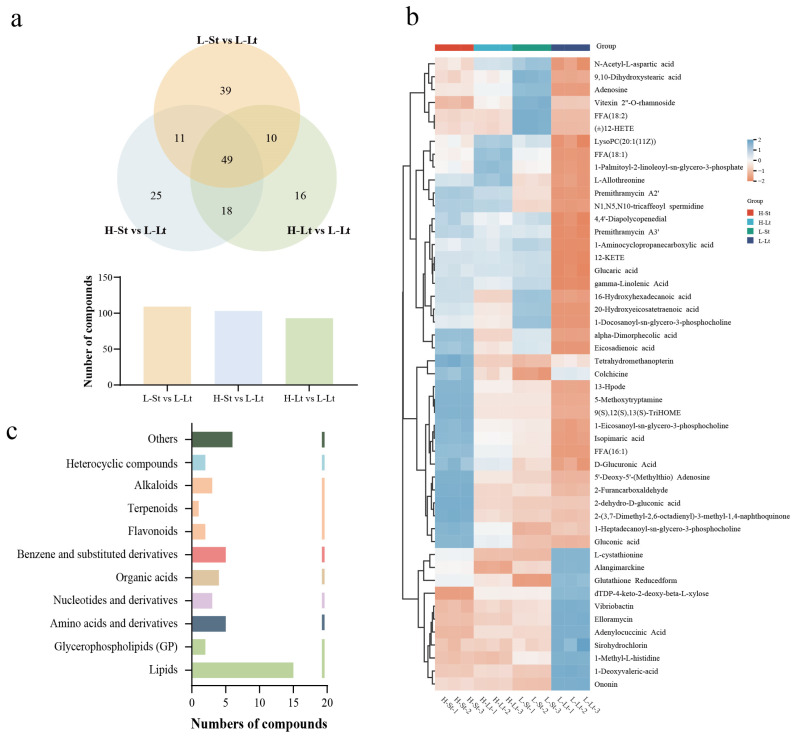
Identification and classification of key metabolites in *S. xylocarp* seeds under different storage conditions: (**a**) Venn diagram of differential accumulated metabolites comparing different storage conditions, (**b**) heatmap of 49 co-expressed differential metabolites, (**c**) classification of 49 co-expressed differential metabolites.

Using a Venn diagram, similarities and intersections among various metabolites across different groups were illustrated (Figure 6a). The results revealed 49 co-expressed differential metabolites, including 11 metabolites showing an upregulation trend and 33 metabolites showing a downregulation trend (Table S2). A cluster analysis based on these foundational data enhanced the visualization of differential metabolites in *S. xylocarpa* seeds. The displayed heatmap visualizes the relative abundance of metabolites in *S. xylocarpa* seeds; the intensity of color in heatmap blocks corresponds to metabolite abundance (Figure 6b). Notably, the lipid compounds are the main metabolites among 49 co-expressed differential metabolites (Figure 6c). Under L-Lt storage conditions, compounds such as 9(S), 12(S), 13(S)-TriHOME, 13-Hpode, and LysoPC(20:1(11Z)) exhibited a downward trend. Conversely, many substances that showed an upward trend under H-St storage conditions were oxidative substances. These findings further confirm that the storage conditions with low seed moisture content and low storage temperatures can suppress the basal metabolic rate of *S. xylocarpa* seeds and better maintain membrane fluidity and integrity.

#### 3.4.2. Analysis of the Important Metabolites and Metabolic Pathways

**Figure 7:**
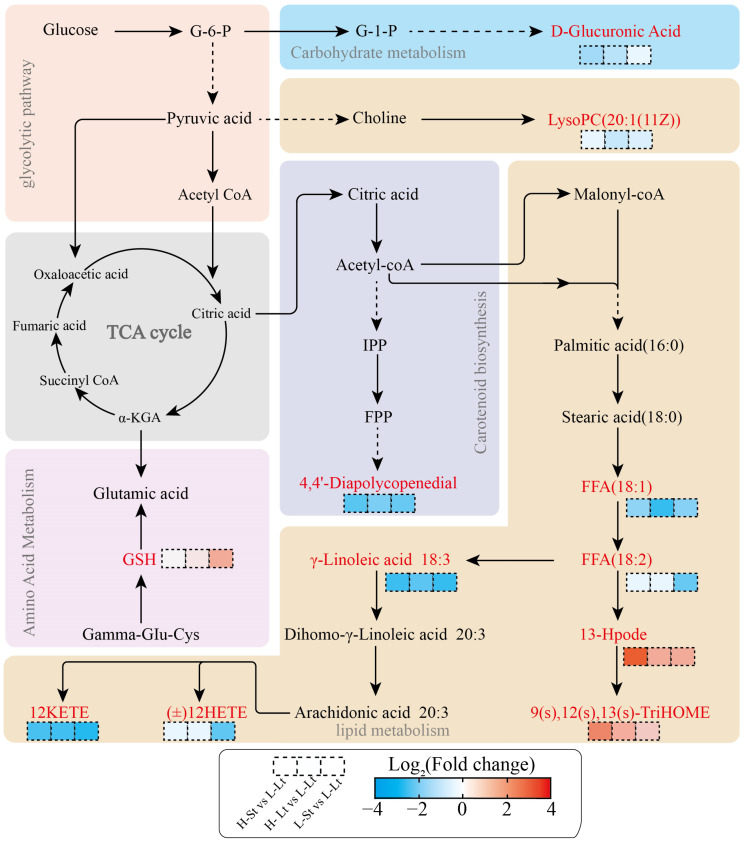
The important metabolites and metabolic pathways in an interrelationship map.

**Figure 8:**
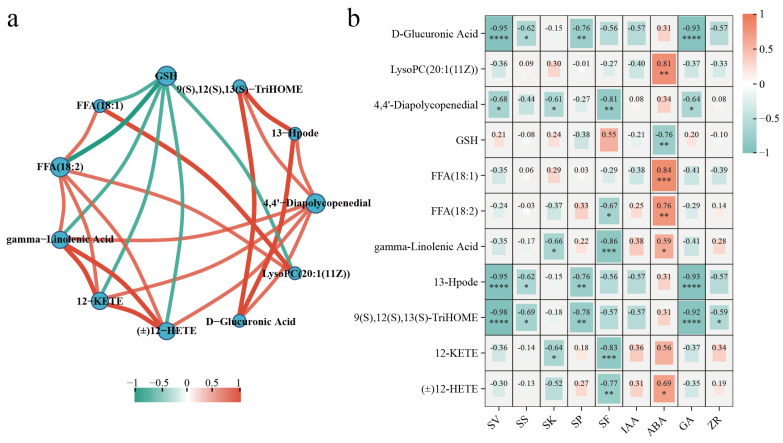
Correlation analysis of key metabolites: (**a**) correlation coefficient network, (**b**) Spearman’s correlation analysis between physiological indicators and key metabolites. According to Spearman’s rank correlation coefficient, (*) *p* < 0.05, (**) *p* < 0.01, (***) *p* < 0.001, (****) *p* < 0.0001. SV, seed viability; SS, soluble sugar; SK, soluble starch; SP, soluble protein.

The chart below involves multiple metabolic pathways, including lipid metabolism, amino acid metabolism, carbohydrate metabolism, and the carotenoid metabolism pathway (Figure 7). We found that, compared to L-Lt, compounds such as FFA (18:1) and FFA (18:2) were significantly downregulated in lipid metabolism, indicating that lipid metabolism was inhibited. Additionally, 4,4′-Diapolycopene-dial and D-Glucuronic Acid were also significantly downregulated. In contrast, the level of GSH, 9(S),12(S),13(S)-TriHOME and 13-Hpode was significantly upregulated compared to L-Lt, which might have been due to stronger oxidative stress under these conditions. To measure the correlations between key metabolites and further understand the interactions among metabolites during seed storage, a correlation coefficient network was constructed using Spearman’s correlation coefficient. In the comparisons of H-St vs. L-Lt, H-Lt vs. L-Lt, and L-St vs. L-Lt, the positive correlations among these key metabolites were significantly higher than the negative correlations (Figure 8a). To further explore the relationship between *S. xylocarpa* seed quality and key metabolites, Spearman’s correlation analysis was performed by calculating Spearman’s correlation coefficients. The correlation heatmap showed that 9(S),12(S),13(S)-TriHOME, 13-Hpode, 4,4′-Diapolycopenedial, and D-Glucuronic Acid were significantly negatively correlated with the seeds’ quality. Additionally, other physiological indicators exhibited sporadic and significant correlations with key metabolites (Figure 8b). These results indicate that reducing the seed moisture content and storage temperature can affect the longevity of *S. xylocarpa* seeds during storage by regulating the lipid metabolism, amino acid metabolism, carbohydrate metabolism, and carotenoid biosynthesis pathways.

## 4. Discussion

### 4.1. Changes in Seed Quality During Storage

For *S. xylocarpa* seeds, which are difficult to germinate naturally, it is challenging to quickly assess seed quality through germination rates. However, the TTC method is not affected by the degree of seed dormancy. The TTC method was used to measure the viability of *Eriochloa villosa* seeds during 8 years of storage; it was found that the staining results were consistent with the germination results [24]. In this study, we observed that the viability of *S. xylocarpa* seeds significantly decreased over the storage period, which is consistent with previous findings [25,26]. Seeds generally exhibit a gradual decline in viability during long-term storage [27]. The key factors influencing seed viability during storage include the seed moisture content and storage temperature [28]. Seeds deteriorate more slowly under conditions of low seed moisture and low storage temperatures. Our study found that the storage effect under the L-Lt condition was significantly better than the effects of other conditions throughout the storage period. This indicates that maintaining a low seed moisture content and storing seeds in a low-temperature environment are highly effective for preserving the quality of *S. xylocarpa* seeds. Additionally, we observed that, in the fresh endosperm cells of *S. xylocarpa* seeds, the organelles were well defined and rich in wax droplets, consistent with previous research [2]. Notably, under the H-St condition, plasmolysis was clearly observed, along with the aggregation of numerous high-electron-density substances. The accumulation of these high-density electron particles suggests that the metabolic processes in *S. xylocarpa* seeds may have been impaired by free radical damage and lipid peroxidation under the H-St storage condition. Furthermore, as the seed moisture content and storage temperature decreased, the organelle structures became more distinct. This indicates that lowering the storage temperature and adjusting the seed moisture content positively contribute to maintaining the viability of *S. xylocarpa* seeds.

### 4.2. Changes in Nutritional Components during Seed Storage

During seed storage, soluble sugars, proteins, fats, and starches serve as primary respiratory substrates, and they play critical roles in maintaining seed viability and cellular membrane stability [29]. Previous studies have shown that, as the seed storage duration increases, the content of soluble sugars decreases, protein synthesis declines, and unsaturated fatty acids undergo continuous oxidation, leading to diminished seed viability [30]. In our study, we found that the soluble carbohydrate content in *S. xylocarpa* seeds stored for 300 days was lower than at the beginning of storage, which is consistent with the findings of Wang [31]. This suggests that the continuous decline in seed viability can be attributed to the depletion of nutritional reserves. Interestingly, we observed a continuous increase in soluble sugar content during storage. This may be due to the breakdown of high-molecular-weight polysaccharides, such as starch, providing substrates and energy for respiratory metabolism. The hydrolysis of starch leads to an increase in glucose content, and soluble sugars serve not only as a primary source of carbon and energy but also as crucial reactive oxygen species (ROS) metabolites. They play a key role in maintaining cellular osmotic pressure and membrane stability [32]. Therefore, the increase in soluble sugar content has a positive effect on storage outcomes. By the end of the storage period, *S. xylocarpa* seeds stored under L-Lt storage conditions contained more soluble carbohydrates. This is mainly because the physiological and biochemical metabolism in seeds under L-Lt storage conditions is reduced, thereby preserving more nutritional substances.

### 4.3. Changes in Endogenous Hormones during Seed Storage

Plant hormones, including ABA, GA_3_, IAA, ZR, and ethylene, are signal molecules that can profoundly influence plant development, even at extremely low concentrations [33]. It has been reported that ABA, IAA, and GA_3_ play crucial roles in seed longevity [34]. Seeds of ABA-insensitive mutants often exhibit reduced dormancy and extended seed longevity [35]. Bueso found that GA can counteract seed deterioration [36]. In our study, the seeds stored under L-Lt storage conditions had significantly higher GA_3_ content throughout the storage period compared to other storage conditions. This result indicates that, under low-storage-temperature and low-seed-moisture conditions, *S. xylocarpa* seeds prolong their lifespan by increasing the GA_3_ content and simultaneously decreasing the ABA content, consistent with findings in rice storage studies [37]. IAA enhances seed longevity by inducing the expression of longevity-related genes and the master regulator ABI3 [38]. In our study, *S. xylocarpa* seeds stored under low-storage-temperature and low-seed-moisture conditions had higher IAA content. Similarly, seed moisture content and storage temperature significantly influenced the ZR content in seeds. Generally, the higher the moisture content and temperature, the lower the seed viability and ZR content.

### 4.4. Prominent Metabolites and Metabolic Pathway Responses of S. xylocarpa Seeds under Different Storage Conditions

Previous metabolomic studies have indicated that rice seeds with poor storability typically contain higher amounts of metabolites, especially those related to amino acids and carbohydrates [39]. In this study, significant metabolic changes might have occurred within the seeds of *S. xylocarpa* under different storage conditions. We speculate that these metabolic changes could be associated with a reduction in seed vigor. Identifying these differential metabolites will help elucidate the molecular mechanisms through which low-temperature and low-moisture storage conditions preserve the longevity of *S. xylocarpa* seeds.

#### 4.4.1. Lipid Metabolism

In the context of lipid metabolism, fatty acids not only serve as a primary energy source in plants but also play crucial roles in physiological regulation [40]. Unsaturated fatty acids, in particular, enhance plant resilience to stress and diseases while maintaining cell membrane integrity [41]. However, the presence of high levels of saturated fatty acids leads to the rapid aging of wheat (*Triticum aestivum*) during storage [42]. This study found that lipid metabolism is a major metabolic pathway during seed storage, with linoleic acid metabolism being significantly enriched across all comparisons. Therefore, our research focuses on understanding how lipid metabolism affects the seed quality and longevity of *S. xylocarpa.*

Linoleic acid is an important unsaturated fatty acid involved in maintaining the structure and function of cell membranes, and it also serves as a precursor to signaling molecules [43]. It is noteworthy that the metabolism of linoleic acid typically produces linoleic acid hydroperoxides (HPODE), which are cytotoxic and capable of damaging important cellular structures such as membranes and nucleic acids, thereby affecting cellular functions [44]. In this study, under the H-St storage conditions, the relative accumulation of hydroperoxide (13-Hpode) was significantly higher compared to other storage conditions (Figure 6b). This indicates that elevated seed moisture content and storage temperature promote HPODE accumulation in *S. xylocarpa* seeds, accelerating seed aging. Similar changes have been reported in wheat during storage, where higher storage temperatures lead to an increased accumulation of HPODE, resulting in cell death and grain deterioration [45]. According to reports, unsaturated fatty acids such as FFA(18:2), FFA(18:1), and γ-Linoleic Acid play multiple roles in seeds. They are not only important components of cell membranes that provide energy reserves but also positively impact plant resistance to environmental stress [46,47]. It is noteworthy that these compounds were significantly downregulated in comparison with the L-Lt condition (Figure 7). This suggests that storage conditions with low seed moisture content and low storage temperatures can reduce oxidative damage and enhance seed adaptability and resilience, thereby prolonging a seed’s lifespan. Additionally, arachidonic acid metabolism, as an important lipid metabolism pathway, plays multiple crucial roles in plant seeds [48]. Among its important metabolic products are 20-Hydroxyeicosatetraenoic Acid (20-HETE) and (±)12-Hydroxy-eicosatetraenoic Acid ((±)12-HETE), which mainly participate in regulating defense mechanisms and adapting to environmental stress [49,50]. In this study, 20-HETE and (±)12-HETE were significantly downregulated (Figure 7), indicating that low-seed-moisture and low-temperature environments help extend the longevity of *S. xylocarpa* seeds. Lysophosphatidylcholine (LysoPC), as one of the important lipid metabolic products, performs the function of repairing and remodeling cell membranes [51]. In this study, LysoPC(20:1(11Z)) was downregulated in all three comparisons, further confirming that reducing storage temperature and seed moisture content can maintain the structural stability of *S. xylocarpa* seeds.

In general, lipid metabolism is an important metabolic process for *S. xylocarpa* seeds during storage, playing a critical role in maintaining the structural stability of cell membranes and cell walls and in resisting environmental stress. Specifically, the reduction in 13-Hpode and the increase in compounds such as 20-HETE, (±)12-HETE, LysoPC(20:1(11Z)) and FFA (18:1) contribute to delaying the aging process of *S. xylocarpa* seeds.

#### 4.4.2. Amino Acid Metabolism

Amino acid biosynthesis plays a crucial role in plant responses to environmental stress. Studies have shown that many defense compounds in plants originate from amino acid precursors [52]. A typical example is the metabolite glutathione reduced form (GSH) in the cysteine and methionine metabolism pathway, which serves as an important antioxidant in plant cells. GSH helps eliminate free radicals and peroxides, protecting cells from oxidative damage [53]. It is noteworthy that, compared to the L-Lt storage condition, there is an upregulation trend for GSH under other storage conditions (Figure 7). We speculate that this increase results from oxidative stress faced by *S. xylocarpa* seeds under higher moisture and storage temperature conditions. The cysteine and methionine metabolism pathway aids seeds in resisting oxidative damage through the glutathione system. Recent studies have also found that wheat enhances glutathione levels to counteract seed aging [45].

#### 4.4.3. Carbohydrate Metabolism

Carbohydrates are essential components of plants, participating in metabolism and structural formation. Nucleotide sugars are complex compounds formed via the combination of sugars and nucleotides (such as UTP, GDP, etc.), playing crucial roles in various biological processes within plants [54]. The biosynthesis pathway of nucleotide sugars involves the conversion of simple sugars into various nucleotide sugars, which are further utilized in the synthesis of polysaccharides, glycoproteins, glycolipids, and other important molecules [55]. D-Glucuronic Acid serves as a significant intermediate and precursor in the biosynthesis pathway of nucleotide sugars. In this study, compared to the L-Lt storage condition, D-Glucuronic Acid was significantly downregulated in the other three storage conditions (Figure 7). The reduced level of D-Glucuronic Acid under these conditions suggests poorer stress resistance of the seeds, further confirming that low storage temperatures and low seed moisture inhibit the physiological activity of *S. xylocarpa* seeds.

#### 4.4.4. Carotenoid Biosynthesis

Carotenoids are a class of plant pigments that play important roles in various physiological processes. As antioxidants, carotenoids protect plant cells from oxidative damage by neutralizing free radicals and ROS [56]. Our study revealed that, compared to the L-Lt storage condition, the levels of 4,4′-Diapolycopenedial were consistently downregulated in all other storage conditions, suggesting a potential inhibition of carotenoid synthesis. Additionally, the downregulation of 4,4′-Diapolycopenedial is negatively correlated with the viability of *S. xylocarpa* seeds (Figure 8). This phenomenon further confirms that low-storage-temperature and low-seed-moisture storage conditions enhance the seed’s ability to neutralize ROS.

## 5. Conclusions

In this study, we observed a significant decline in the viability of *S. xylocarpa* seeds during storage. However, when the seeds were stored under conditions of low seed moisture content and low storage temperature (L-Lt), the preservation effect was significantly better than under other storage conditions. Specifically, under this storage condition, seed viability, soluble sugars, soluble proteins, and GA_3_ content reached their highest levels, and organelle structures remained the most intact. Additionally, we identified 335 known metabolites in *S. xylocarpa* seeds, with lipids, organic acids, and secondary metabolites being the three most abundant classes. Notably, under optimal storage conditions, *S. xylocarpa* seeds delayed aging and maintained seed viability by upregulating protective metabolites such as GSH, LysoPC (20:1(11Z)), 20-HETE, and (±)12-HETE while downregulating harmful hydroperoxide like 13-HpODE. These processes involved the regulation of lipid, amino acid, carbohydrate, and carotenoid metabolic pathways. However, these conclusions are based on metabolomic analysis alone, and further molecular studies are required to elucidate how low-temperature and low-seed-moisture conditions specifically extend the lifespan of *S. xylocarpa* seeds.

## Data Availability

The original contributions presented in the study are included in the article/Supplementary Material, further inquiries can be directed to the corresponding author.

## Supplementary Materials

The following supporting information can be downloaded at https://www.mdpi.com/article/10.3390/metabo14090503/s1: Table S1: All detected metabolites for wholegrain flour all the metabolites identified via KEGG can be detected using *S. xylocarpa* seeds; Table S2: Differential expression of key metabolites in *S. xylocarpa* seeds under different storage condition.
