# Supplementary material for: Metabolomic and Physiological Analyses Reveal the Effects of Different Storage Conditions on Sinojackia xylocarpa Hu Seeds
Source: Metabolites. 2024 Sep 18;14(9):503. doi: 10.3390/metabo14090503 (PMC11434619; doi:10.3390/metabo14090503)
Supplement: Supplementary file 1 [file metabolites-14-00503-s001.zip › metabolites-3176468-supplementary.pdf]

Supporting Information

Metabolomic and physiological analyses reveal the effects of different storage conditions on *Sinojackia xylocarpa* Hu seeds

Hao Cai and Yongbao Shen*

Collaborative Innovation Centre of Sustainable Forestry in Southern China, College of Forestry, Nanjing Forestry University, Nanjing 210037, China

*Correspondence author: Yongbao Shen

E-mail addresses: ybshen@njfu.edu.cn

Table. S1 All detected metabolites for wholegrain flour all the metabolites identified by KEGG can be detected by the *S. xylocarpa*. Seeds.

Compounds	Classificat	H-St	H-Lt	L-St	L-Lt	Cpd_
N-Acetyl-L-aspartic acid	Amino	2.91E+	3.85E+	4.41E+	1.57E+	C010
5-Formyl-5,6,7,8-	Alkaloids	6.32E+	9.42E+	1.01E+	9.93E+	C012
Allantoic acid	Organic	4.44E+	4.63E+	5.65E+	4.49E+	C004
Pyroglutamic acid	Amino	2.10E+	1.80E+	1.34E+	2.05E+	C018
2-Furancarboxaldehyde	Others	1.89E+	5.41E+	6.20E+	2.34E+	C142
9(S),12(S),13(S)-TriHOME	Lipids	6.93E+	3.00E+	2.94E+	9.86E+	C148
Methyl (+)-7-isojasmonate	Organic	4.42E+	4.03E+	3.47E+	3.30E+	C163
2'-Deoxycytidine	Nucleotide	5.15E+	5.16E+	5.66E+	4.63E+	C008
2-Aminophenol	Alkaloids	2.63E+	2.67E+	2.59E+	2.57E+	C019
PA(P-16:0/18:2(9Z,12Z))	GP	5.13E+	8.43E+	2.86E+	5.34E+	C156
Glc4Me(a1-4)Glc(a)-O-Me	Alcohol	1.82E+	1.64E+	1.47E+	1.69E+	C007
2'-Deoxyinosine	Nucleotide	1.23E+	1.10E+	1.55E+	2.30E+	C055
Vibriobactin	Benzene	4.24E+	5.62E+	5.91E+	1.20E+	C067
Indole-3-acetamide	Alkaloids	7.12E+	1.60E+	8.52E+	1.56E+	C026
1-(9Z-hexadecenoyl)-sn-glycero-3-	Lipids	1.19E+	7.43E+	1.07E+	6.63E+	C042
Sphinganine	SL	1.28E+	9.04E+	5.72E+	8.15E+	C008
1-O-Octadecyl-SN-glycero-3-	GP	3.56E+	2.05E+	3.98E+	2.40E+	C043
Betaine aldehyde	Others	5.15E+	7.32E+	4.63E+	7.54E+	C005
Sinapic acid	Phenolic	7.01E+	4.07E+	1.53E+	5.83E+	C004
L-Isoleucine	Amino	4.09E+	4.19E+	2.10E+	4.59E+	C004
Cytosine	Nucleotide	5.21E+	1.84E+	3.22E+	2.13E+	C003
L-Histidine	Amino	5.51E+	6.78E+	7.42E+	9.33E+	C001
1,2-Dipalmitoyl-sn-glycerol 3-	GP	6.52E+	6.89E+	4.53E+	3.95E+	C004
(S)-10,16-Dihydroxyhexadecanoic	Lipids	1.35E+	1.45E+	1.18E+	8.11E+	C082
Pulegone	Terpenoid	2.37E+	2.18E+	2.41E+	2.70E+	C098
Thiourocanic acid	Organic	8.54E+	8.12E+	9.16E+	8.05E+	C055
L-Homoserine	Amino	3.48E+	3.70E+	2.57E+	2.19E+	C002
5-Aminopentanoic acid	Organic	1.82E+	2.12E+	2.16E+	2.42E+	C004
2'-Deoxyadenosine-5'-	Nucleotide	3.54E+	3.12E+	4.83E+	5.42E+	C003
Sirohydrochlorin	Heterocycl	1.41E+	1.53E+	1.78E+	3.20E+	C057
Nicotinic acid adenine dinucleotide	Nucleotide	3.27E+	2.53E+	1.75E+	3.22E+	C008
D-Fructose-6-phosphate	Others	1.40E+	1.16E+	5.48E+	8.99E+	C000
Traumatic acid	Lipids	2.45E+	2.32E+	2.40E+	2.64E+	C163
Cytidine 2',3'-cyclic	Nucleotide	4.49E+	7.76E+	1.47E+	5.15E+	C023
L-Phenylalanine	Amino	2.35E+	3.70E+	1.80E+	3.10E+	C000
Colfosceril palmitate	GP	1.13E+	2.06E+	6.30E+	2.89E+	C001
Methionine	Amino	6.30E+	7.28E+	6.77E+	7.37E+	C000
5-methylthio-D-ribose	Others	1.43E+	1.66E+	1.75E+	1.30E+	C030
Choline	Alkaloids	1.62E+	2.50E+	3.90E+	2.05E+	C001
Dopamine	Alkaloids	1.69E+	1.87E+	2.71E+	1.86E+	C037
N-Methylhydantoin	Heterocycl	2.75E+	2.29E+	2.53E+	3.61E+	C025
2-Hydroxycinnamic acid	Phenolic	1.50E+	1.35E+	1.26E+	1.45E+	C017
Adenosine monophosphate	Nucleotide	5.31E+	4.12E+	5.06E+	3.80E+	C000
5-Methoxytryptamine	Alkaloids	1.73E+	7.51E+	7.49E+	2.07E+	C056
Aminocaproic acid	Organic	5.05E+	3.78E+	2.90E+	3.77E+	C023

L-Tyrosine	Amino	2.68E+	2.36E+	2.21E+	2.58E+	C000
8-Methyl-6-nonenoic acid	Organic	1.61E+	1.53E+	1.28E+	1.95E+	C182
Folic acid	Others	1.31E+	1.09E+	1.19E+	1.49E+	C005
Isopimaric acid	Terpenoid	8.59E+	4.86E+	4.30E+	9.51E+	C091
Matairesinol	Lignans	7.68E+	8.13E+	8.07E+	9.19E+	C106
D-Xylose	Others	8.90E+	1.00E+	8.99E+	9.54E+	C001
Caffeine	Alkaloids	6.53E+	4.17E+	5.85E+	4.05E+	C074
Isoalangiside	Benzene	5.52E+	7.26E+	5.53E+	7.02E+	C118
Sucrose	Others	3.85E+	2.31E+	2.51E+	3.01E+	C000
L-Glutamic acid	Amino	2.36E+	3.25E+	2.56E+	2.58E+	C000
Glutamine	Amino	1.78E+	1.21E+	7.48E+	6.67E+	C000
beta-D-gentiobiosyl crocetin	Terpenoid	2.65E+	4.55E+	4.13E+	1.96E+	C198
Phytosphingosine	Lipids	6.52E+	6.66E+	5.25E+	4.80E+	C121
Agroclavine	Alkaloids	5.90E+	9.45E+	3.96E+	9.91E+	C090
Taurocyamine	Organic	4.12E+	4.64E+	4.24E+	4.65E+	C019
Ferulic acid	Phenolic	2.10E+	9.75E+	9.03E+	9.92E+	C014
Pipecolic acid	Alkaloids	3.41E+	3.47E+	3.30E+	3.92E+	C004
Pyridoxamine phosphate	Organic	1.72E+	2.02E+	2.37E+	2.04E+	C006
D-Xylonic acid	Others	1.53E+	1.73E+	1.65E+	1.50E+	C005
Abietic acid	Terpenoid	3.39E+	1.79E+	1.01E+	4.03E+	C060
Histamine	Alkaloids	6.53E+	4.16E+	4.16E+	3.71E+	C003
1-Heptadecanoyl-sn-glycero-3-	Lipids	2.69E+	1.37E+	2.36E+	5.94E+	C042
gamma-Linolenic Acid	Lipids	2.20E+	1.95E+	2.28E+	3.99E+	C064
4-Hydroxycinnamic acid	Phenolic	1.16E+	8.32E+	6.52E+	8.64E+	C008
Thymidine	Nucleotide	7.77E+	1.10E+	1.71E+	1.60E+	C002
Ergothioneine	Alkaloids	3.83E+	4.89E+	4.43E+	3.21E+	C055
5-Ureidoimidazole-4-carboxylic	Organic	2.77E+	2.66E+	3.54E+	2.18E+	C055
5-Aminoimidazole	Heterocycl	8.47E+	8.54E+	6.67E+	6.73E+	C052
Harman	Alkaloids	2.06E+	2.11E+	1.97E+	1.80E+	C092
Reticuline	Alkaloids	2.18E+	1.87E+	5.89E+	4.06E+	C021
Deoxyuridine-5'-diphosphate	Nucleotide	1.69E+	4.26E+	3.08E+	3.23E+	C013
5-Aminoimidazole-4-carboxamide	Alcohol	9.20E+	9.83E+	8.17E+	8.50E+	C040
Isopentenyl pyrophosphate	Organic	1.55E+	1.58E+	1.61E+	1.49E+	C001
Cytidine diphosphate ribitol	Nucleotide	3.77E+	3.54E+	2.99E+	5.62E+	C007
1-Methyl-L-histidine	Amino	6.62E+	6.49E+	9.99E+	1.80E+	C011
L-methionine sulfoxide	Amino	4.90E+	5.60E+	4.63E+	7.00E+	C029
Guanosine diphosphate mannose	Nucleotide	1.45E+	1.92E+	1.29E+	2.06E+	C000
Agmatine	Alkaloids	1.03E+	1.26E+	9.46E+	1.45E+	C001
Homovanillic acid	Phenolic	3.44E+	3.34E+	3.62E+	3.81E+	C055
6-Demethylsterigmatocystin	Heterocycl	4.13E+	1.28E+	8.90E+	3.66E+	C036
2-Pyrocatechuic acid	Phenolic	6.64E+	6.24E+	4.20E+	3.23E+	C001
4-Acetamidobutanoic acid	Organic	3.23E+	3.17E+	2.74E+	3.81E+	C029
3-Dehydroshikimate	Organic	1.90E+	4.15E+	3.45E+	3.54E+	C026
Oxiglutatione	Amino	5.08E+	2.73E+	2.18E+	1.74E+	C001
Aerobactin	Amino	4.71E+	4.21E+	3.82E+	6.27E+	C055
5-Aminoimidazole ribonucleotide	Nucleotide	1.37E+	1.20E+	1.03E+	1.57E+	C033
4-Hydroxy-l-tryptophan	Amino	1.15E+	1.10E+	1.18E+	1.46E+	C217
L-Allothreonine	Amino	6.17E+	7.40E+	4.56E+	2.71E+	C055
Neurosporaxanthin	Terpenoid	9.58E+	4.94E+	1.90E+	5.88E+	C086

5'-Deoxy-5'-(Methylthio)	Nucleotide	2.65E+	8.44E+	5.47E+	2.19E+	C001
13-Hpode	Lipids	2.89E+	1.35E+	1.14E+	1.99E+	C047
1-Aminocyclopropanecarboxylic	Organic	3.27E+	3.40E+	3.98E+	5.09E+	C012
D-(+)-Neopterin	Alkaloids	1.49E+	1.37E+	1.17E+	1.30E+	C059
Docosadienoate (22:2n6)	Lipids	1.90E+	2.10E+	2.75E+	2.02E+	C165
Floionolic acid	Lipids	1.05E+	2.56E+	2.50E+	3.37E+	C196
Nicotiflorin	Flavonoid	2.42E+	2.02E+	7.29E+	1.45E+	C218
LysoPC 16:0	Lipids	9.95E+	9.66E+	1.12E+	7.05E+	C042
Arachidonyl-coa	FA	2.96E+	1.37E+	2.33E+	3.86E+	C022
Trans-Zeatin	Alkaloids	5.02E+	6.79E+	6.88E+	8.55E+	C003
N-Acetyl-L-glutamyl 5-phosphate	Organic	9.94E+	1.16E+	8.87E+	1.24E+	C041
Mevalonate	Organic	1.57E+	1.11E+	1.64E+	1.29E+	C004
Erythritol	Others	5.58E+	5.56E+	7.21E+	6.60E+	C005
L-Proline	Amino	1.26E+	1.18E+	1.11E+	1.36E+	C001
Alangimarckine	Alkaloids	6.65E+	3.45E+	5.45E+	1.05E+	C093
4a-Hydroxytetrahydrobiopterin	Others	6.48E+	5.15E+	7.56E+	5.72E+	C155
Nicotinamide riboside	Alcohol	1.35E+	1.56E+	1.38E+	1.55E+	C031
(S)-7-(((2-O-6-Deoxy-alpha-L-	Flavonoid	4.26E+	3.35E+	1.82E+	1.55E+	C098
Acetyl adenylate	Nucleotide	3.84E+	1.04E+	8.56E+	8.69E+	C059
4-Isopropylbenzaldehyde	Others	7.15E+	8.88E+	1.06E+	1.01E+	C065
Vanillylamine	Alkaloids	2.44E+	2.34E+	2.03E+	2.76E+	C166
2-(Methylamino)benzoic acid	Benzene	1.62E+	3.26E+	1.15E+	1.14E+	C030
2-Methylbenzoic acid	Phenolic	1.34E+	1.12E+	1.02E+	1.29E+	C072
Maltol	Heterocycl	6.21E+	6.39E+	5.82E+	6.49E+	C119
Guanosine	Nucleotide	5.77E+	6.86E+	9.27E+	5.72E+	C003
2-(3,7-Dimethyl-2,6-octadienyl)-3-	Benzene	2.30E+	4.96E+	3.14E+	1.09E+	C008
Emindole Sb	Alkaloids	2.67E+	1.94E+	6.33E+	3.19E+	C205
Piperidine	Alkaloids	3.95E+	3.00E+	2.36E+	3.01E+	C017
Nicotinamide mononucleotide	Nucleotide	1.53E+	1.66E+	2.93E+	1.74E+	C004
1-Eicosanoyl-sn-glycero-3-	Lipids	1.31E+	6.97E+	6.62E+	1.49E+	C042
Betaine	Alkaloids	1.75E+	7.75E+	6.66E+	6.22E+	C007
Naringin	Flavonoid	1.47E+	1.65E+	1.32E+	4.83E+	C097
Triethanolamine	Alcohol	5.69E+	7.65E+	6.64E+	6.75E+	C067
1-Palmitoyl-2-linoleoyl-sn-glycero-	GP	5.54E+	8.20E+	5.33E+	2.37E+	C004
1-Palmitoyl-2-oleoyl-sn-glycero-3-	GP	2.45E+	4.78E+	2.61E+	1.28E+	C004
1-Pentadecanoyl-sn-glycero-3-	GP	2.13E+	1.05E+	2.23E+	1.53E+	C042
3,3'-Bipyridine-2,2',5,5',6,6'-hexol	Heterocycl	1.18E+	1.72E+	2.28E+	2.29E+	C203
2-Benzylmalate	Organic	2.97E+	2.60E+	3.13E+	3.03E+	C206
NE,NE,NE-TRIMETHYLLYSINE	Amino	7.04E+	1.20E+	5.57E+	6.63E+	C037
(3R)-9-[(2R,4R,5S,6R)-4,5-	Benzene	3.66E+	2.54E+	4.46E+	3.66E+	C124
3-Methoxytyramine-betaxanthin	Phenolic	8.83E+	8.81E+	9.39E+	1.09E+	C177
2-(2-Carboxy-4-methylthiazol-5-	Organic	5.75E+	9.39E+	1.20E+	1.09E+	C202
(1S,4aS,7aS)-7-methyl-1-	Terpenoid	3.57E+	3.32E+	3.92E+	4.14E+	C116
(2R,3R)-3-Methylglutamyl-5-	Organic	1.70E+	2.27E+	2.38E+	2.41E+	C202
1-Stearoyl-2-linoleoyl-sn-glycero-	GP	4.48E+	3.58E+	3.80E+	3.06E+	C004
6'-Oxokanamycin X	Heterocycl	3.25E+	1.88E+	2.29E+	2.60E+	C212
Glutathione Reducedform	Amino	6.08E+	4.64E+	5.01E+	1.02E+	C000
1D-3-amino-1-guanidino-1,3-	Alcohol	3.29E+	2.93E+	2.94E+	3.00E+	C012
Ureidoacrylic acid	Organic	3.25E+	2.60E+	2.39E+	3.38E+	C202

LysoPC(20:1(11Z))	Lipids	3.22E+	4.04E+	3.51E+	2.01E+	C042
(3R)-9-[(2R,4R,5R,6R)-4-	Benzene	8.80E+	7.52E+	7.32E+	6.85E+	C124
N1,N5,N10-tricaffeoyl spermidine	Heterocycl	1.37E+	1.29E+	5.93E+	4.38E+	C180
1-Deoxyvaleric-acid	Organic	1.18E+	1.30E+	1.17E+	2.21E+	C204
2-Amino-4-[(3-	Amino	1.52E+	1.64E+	1.10E+	1.73E+	C181
Biuret	Others	1.06E+	5.91E+	6.09E+	5.05E+	C065
(S)-2-acetamido-6-oxopimelic acid	Organic	8.24E+	4.98E+	8.58E+	6.01E+	C055
N6,N6,O-Tridemethylpuromycin-	Nucleotide	6.30E+	2.31E+	6.09E+	4.44E+	C070
Galabiosylceramide	SL	4.04E+	3.25E+	2.96E+	9.08E+	C061
5-Oxo-4,5-dihydro-2-furylacetic	Organic	1.40E+	1.31E+	1.36E+	9.59E+	C035
2-Descarboxy-betanidin	Organic	2.36E+	2.10E+	2.88E+	2.94E+	C177
(13E)-labda-7,13-dien-15-ol	Alcohol	1.91E+	3.49E+	1.27E+	3.12E+	C201
3-	Organic	2.19E+	2.05E+	3.48E+	4.45E+	C063
1-Hydroxy-13-O-acetyl-N-	Alkaloids	5.71E+	6.57E+	5.32E+	3.85E+	C215
Premithramycin A3	Benzene	6.14E+	7.68E+	5.05E+	7.90E+	C123
9-((2R,3R,4S,5R)-3,4-Dihydroxy-	Nucleotide	2.08E+	2.10E+	1.20E+	2.08E+	C163
1-Guanidino-1-deoxy-scyllo-	Alcohol	5.78E+	5.27E+	6.41E+	4.22E+	C042
1,2-Dipalmitoleoyl-sn-glycero-3-	GP	6.95E+	9.18E+	2.97E+	9.23E+	C001
alpha-1,5-L-Arabinotetraose	Others	1.48E+	1.44E+	1.62E+	1.85E+	C205
glandicoline A	Others	5.60E+	4.78E+	3.56E+	7.02E+	C221
Premithramycin A2'	Benzene	8.27E+	7.65E+	5.87E+	3.90E+	C123
(4-Amino-2-methylpyrimidin-5-	Organic	3.22E+	2.67E+	2.51E+	2.55E+	C045
4-Methyl-5-hydroxyethylthiazole	Organic	1.03E+	1.24E+	1.89E+	1.32E+	C043
syn-Copalyl diphosphate	Organic	9.87E+	7.50E+	7.37E+	7.91E+	C119
8-demethyl-8-(2,3-O-dimethyl-	Benzene	1.03E+	7.35E+	1.33E+	1.11E+	C209
dTDP-D-fucose	Nucleotide	1.19E+	1.34E+	6.57E+	5.44E+	C072
cyclo-Dopa-glucuronylglucoside	Benzene	1.13E+	9.42E+	8.79E+	1.10E+	C177
LysoPC(22:2(13Z,16Z))	Lipids	2.46E+	8.39E+	7.15E+	3.84E+	C042
5'-Butyrylphosphoinosine	Organic	4.04E+	1.58E+	2.35E+	2.68E+	C064
dTDP-3-amino-2,3,6-trideoxy-C-	Nucleotide	1.40E+	1.63E+	1.66E+	1.92E+	C123
3-(Acetamidomethylidene)-2-	Organic	7.44E+	4.13E+	4.35E+	2.80E+	C046
12-Deoxyaklanonic acid	Benzene	3.01E+	2.76E+	2.61E+	3.47E+	C124
[(1R,2R,3R)-2-methyl-3-[(5E,9E)-	Terpenoid	2.17E+	7.13E+	7.43E+	2.38E+	C034
L-Saccharopine	Amino	2.21E+	2.35E+	2.10E+	2.52E+	C004
Trehalose	Others	5.11E+	5.36E+	5.78E+	5.19E+	C010
CDP-choline	Nucleotide	1.00E+	9.02E+	8.93E+	9.29E+	C003
Elloramycin	Benzene	1.78E+	2.05E+	2.35E+	4.39E+	C123
2'-Deoxyuridine	Nucleotide	2.51E+	2.24E+	2.68E+	3.00E+	C005
5-Methyl-5,6,7,8-	Heterocycl	1.05E+	1.16E+	8.84E+	1.32E+	C044
Tetrahydromethanopterin	Organic	2.77E+	3.94E+	1.13E+	8.00E+	C012
6-Phosphogluconic acid	Others	2.23E+	2.91E+	2.28E+	2.36E+	C003
Gossypol	Terpenoid	1.52E+	1.31E+	7.98E+	9.05E+	C076
Lobeline	Alkaloids	2.32E+	3.97E+	1.27E+	1.09E+	C074
Phthalic acid	Phenolic	9.21E+	9.10E+	8.01E+	7.88E+	C016
Glucaric acid	Others	8.80E+	8.81E+	9.04E+	2.11E+	C008
Tartaric acid	Organic	3.60E+	2.97E+	4.05E+	4.31E+	C008
Gramine	Alkaloids	6.70E+	7.09E+	3.24E+	2.44E+	C083
3'-Aenylic Acid	Nucleotide	3.32E+	2.94E+	3.36E+	4.83E+	C013
Rotenone	Flavonoid	4.49E+	3.55E+	3.49E+	3.69E+	C075

Tetrahydroalstonine	Alkaloids	9.72E+	8.57E+	7.89E+	8.31E+	C116
Ketoleucine	Organic	5.45E+	3.87E+	5.28E+	7.29E+	C002
20-Hydroxyeicosatetraenoic acid	Lipids	3.87E+	2.96E+	4.74E+	1.15E+	C147
3,4-Dihydroxymandelic acid	Organic	1.52E+	1.59E+	1.63E+	1.67E+	C055
Citric acid	Organic	2.56E+	3.17E+	2.77E+	3.39E+	C001
Geranyl diphosphate	Organic	1.07E+	1.53E+	1.74E+	1.79E+	C058
All-trans-heptaprenyl diphosphate	Organic	1.83E+	4.52E+	1.22E+	1.09E+	C042
4-oxo-4-(3-pyridyl)butyraldehyde	Alkaloids	2.17E+	2.17E+	2.33E+	2.06E+	C195
N-Acetyl-D-glucosamine	Alcohol	3.06E+	3.43E+	3.54E+	2.87E+	C001
Arginine	Amino	1.44E+	8.09E+	7.78E+	8.90E+	C000
Mevalonate 5-phosphate	Organic	1.02E+	1.26E+	1.17E+	1.13E+	C011
alpha-Dimorphecolic acid	Lipids	3.44E+	1.32E+	2.43E+	3.05E+	C147
L-Tryptophan	Amino	5.95E+	5.75E+	6.16E+	7.96E+	C000
3-Dihydropyridine-2,6-	Heterocycl	3.13E+	2.01E+	1.66E+	2.39E+	C085
(±)12-HETE	Lipids	9.59E+	9.22E+	2.56E+	5.18E+	C147
Hordatine A	Alkaloids	8.59E+	1.02E+	1.10E+	1.13E+	C083
Xylobiose	Others	8.29E+	1.24E+	1.06E+	1.31E+	C016
Xanthohumol	Flavonoid	7.12E+	8.42E+	8.92E+	9.35E+	C164
D-phenylalanine	Amino	4.21E+	3.94E+	3.93E+	3.75E+	C022
2-dehydro-D-gluconic acid	Others	2.57E+	2.59E+	6.98E+	4.52E+	C064
Cytidine	Nucleotide	9.37E+	1.02E+	9.38E+	1.02E+	C004
Vitexin 2"-O-rhamnoside	Flavonoid	1.50E+	9.72E+	2.44E+	3.97E+	C126
Colchicine	Alkaloids	1.83E+	7.22E+	2.55E+	1.19E+	C075
D-Malic acid	Organic	3.16E+	3.84E+	2.94E+	2.64E+	C004
Uridine triphosphate(UTP)	Nucleotide	1.45E+	1.63E+	6.90E+	1.12E+	C000
Cytidine-5'-diphosphate	Nucleotide	1.54E+	1.76E+	1.80E+	1.88E+	C001
L-Homocystine	Amino	1.35E+	1.16E+	3.20E+	1.68E+	C018
Melilotoside	Benzene	4.59E+	3.50E+	2.73E+	3.24E+	C051
Chorismic acid	Organic	4.24E+	4.40E+	4.65E+	5.09E+	C002
1-Methylxanthine	Nucleotide	6.15E+	6.21E+	5.77E+	6.20E+	C163
Aklavin	Heterocycl	1.00E+	9.44E+	8.56E+	1.15E+	C186
FFA(18:2)	Lipids	9.26E+	9.68E+	2.56E+	5.75E+	C015
Cucurbitacin C	Others	3.36E+	2.91E+	3.77E+	3.45E+	C087
Nicotinuric acid	Amino	2.17E+	2.56E+	2.61E+	2.65E+	C053
Anatabine	Alkaloids	4.80E+	9.14E+	7.98E+	8.81E+	C101
Uridine-5'-monophosphate	Nucleotide	8.26E+	9.84E+	9.23E+	1.07E+	C001
Uridine	Nucleotide	1.77E+	1.47E+	1.35E+	1.58E+	C002
Adenosine 5'-Diphosphate（ADP）	Nucleotide	9.46E+	1.08E+	6.68E+	1.11E+	C000
Inosine	Nucleotide	1.03E+	1.07E+	1.03E+	1.08E+	C002
Adenosine	Nucleotide	4.74E+	5.43E+	7.75E+	2.41E+	C002
D-Allose	Others	1.66E+	1.52E+	2.23E+	1.40E+	C014
Strychnine	Alkaloids	9.46E+	6.67E+	6.70E+	5.49E+	C065
FFA(18:0)	Lipids	1.42E+	1.98E+	1.86E+	1.97E+	C015
FFA(16:0)	Lipids	3.83E+	3.49E+	3.63E+	3.27E+	C002
2-Amino-3-phosphonopropionic	Organic	5.00E+	2.89E+	2.42E+	3.15E+	C056
L-cystathionine	Amino	1.59E+	7.11E+	6.44E+	2.74E+	C022
Eicosadienoic acid	Lipids	1.89E+	1.12E+	1.50E+	4.18E+	C165
FFA(14:0)	Lipids	4.65E+	5.59E+	5.86E+	4.33E+	C064
D-Glucuronic Acid	Others	2.26E+	1.69E+	1.18E+	7.28E+	C001

Galactaric acid	Others	1.10E+	1.41E+	2.29E+	1.63E+	C008
Gluconic acid	Others	1.16E+	6.35E+	1.93E+	1.07E+	C002
Narcotoline	Alkaloids	1.30E+	1.98E+	2.76E+	1.43E+	C095
Raffinose	Others	1.66E+	2.45E+	2.62E+	2.32E+	C004
FFA(20:0)	Lipids	4.17E+	3.48E+	7.71E+	4.83E+	C064
16-Hydroxyhexadecanoic acid	Lipids	2.94E+	1.91E+	3.46E+	1.11E+	C182
(2S)-2-Isopropylmalate	Organic	1.26E+	8.69E+	1.19E+	1.67E+	C025
Pantetheine	Alkaloids	1.09E+	1.03E+	9.55E+	9.88E+	C008
Thiamine monophosphate	Others	1.22E+	1.34E+	1.76E+	1.20E+	C010
Arabinonic acid	Organic	3.59E+	3.88E+	4.45E+	4.52E+	C008
Daidzein	Flavonoid	4.27E+	4.66E+	4.49E+	4.75E+	C102
Ononin	Flavonoid	9.57E+	9.22E+	8.32E+	1.65E+	C105
Podofilox	Lignans	3.02E+	2.35E+	2.16E+	2.52E+	C108
Stachyose	Others	8.55E+	1.38E+	1.40E+	1.33E+	C016
Curcumin	Phenolic	7.04E+	7.24E+	5.22E+	9.32E+	C104
Trehalose-6-phosphate	Others	1.00E+	7.75E+	7.43E+	6.89E+	C006
2-amino-4-oxovaleric acid	Organic	3.81E+	3.73E+	3.00E+	3.25E+	C033
Diguanosine tetraphosphate	Nucleotide	5.81E+	2.79E+	1.16E+	6.22E+	C012
Phosphoserine	Amino	2.75E+	1.20E+	3.08E+	2.70E+	C010
Dihydrofolic acid	Organic	3.51E+	3.63E+	3.68E+	4.78E+	C004
FFA(16:1)	Lipids	2.28E+	1.53E+	1.27E+	4.44E+	C083
Citrulline	Amino	5.54E+	5.72E+	5.60E+	5.44E+	C003
Galactinol	Others	9.83E+	3.35E+	3.71E+	3.90E+	C012
Thymidine-5'-phosphate	Nucleotide	7.40E+	4.50E+	3.11E+	5.93E+	C003
UDP-xylose	Nucleotide	3.36E+	3.98E+	1.58E+	2.50E+	C001
Oxoglutaric acid	Organic	3.35E+	3.38E+	3.40E+	4.23E+	C000
Oxalacetic acid	Organic	1.06E+	6.71E+	9.69E+	1.19E+	C000
D-Glucuronolactone	Others	3.63E+	1.21E+	6.84E+	8.06E+	C026
Oxoadipic acid	Organic	5.53E+	8.18E+	7.68E+	7.84E+	C003
Spaglumic acid	Amino	2.17E+	1.82E+	2.07E+	1.90E+	C122
Novobiocin	Heterocycl	1.41E+	1.24E+	1.04E+	1.13E+	C050
Amygdalin	Alkaloids	2.78E+	3.69E+	4.93E+	6.61E+	C083
2-Hydroxyglutarate	Organic	1.08E+	8.89E+	9.18E+	8.78E+	C026
Choline Alfoscerate	Lipids	1.40E+	1.69E+	2.07E+	1.65E+	C006
Phenylacetylglutamine	Amino	2.40E+	2.50E+	2.70E+	2.63E+	C041
Sedoheptulose 7-phosphate	Others	1.04E+	9.14E+	1.14E+	8.40E+	C053
cis-coumarinic acid-beta-D-	Phenolic	1.17E+	3.00E+	1.53E+	1.84E+	C058
Macarpine	Alkaloids	1.64E+	1.18E+	1.36E+	1.87E+	C061
Dolichyl b-D-glucosyl phosphate	Organic	5.33E+	3.17E+	2.19E+	4.74E+	C012
Strictosidine	Alkaloids	5.50E+	6.59E+	5.47E+	8.59E+	C034
Cysteinylglycine	Amino	5.08E+	4.56E+	4.13E+	4.65E+	C014
Adenylocuccinic Acid	Organic	1.84E+	2.53E+	2.41E+	4.64E+	C037
Loganin	Terpenoid	1.23E+	1.14E+	1.07E+	1.34E+	C014
Chelirubine	Alkaloids	1.26E+	5.17E+	1.70E+	1.41E+	C063
2-benzylmalate	Benzene	5.55E+	1.07E+	1.02E+	3.82E+	C206
D-Mannitol 1-phosphate	Others	4.44E+	4.98E+	6.07E+	5.94E+	C006
Keto-Deoxy-Nonulonic acid	Organic	6.72E+	7.17E+	9.17E+	1.01E+	C209
alpha-D-glucose-6-phosphate	Others	5.27E+	2.72E+	5.05E+	4.09E+	C006
Xanthosine	Nucleotide	1.13E+	1.23E+	9.68E+	1.49E+	C017

Flavin adenine dinucleotide	Nucleotide	2.23E+	1.62E+	1.63E+	2.04E+	C000
D-inositol-4-phosphate	Alcohol	1.44E+	1.97E+	1.77E+	1.93E+	C035
Ginsenoside Rg3	Terpenoid	7.97E+	8.54E+	4.53E+	6.06E+	C207
Fumiquinazoline C	Alkaloids	3.14E+	2.95E+	2.89E+	3.38E+	C221
Uridine-5'-diphosphate-glucose	Nucleotide	9.96E+	1.25E+	4.04E+	1.37E+	C000
Flavin Single Nucleotide(FMN)	Nucleotide	2.47E+	1.89E+	2.78E+	2.62E+	C000
4-Hydroxybenzaldehyde	Phenolic	5.38E+	5.08E+	4.87E+	5.23E+	C006
Quercetin	Flavonoid	6.63E+	6.15E+	6.30E+	8.89E+	C003
L-Gulonolactone	Others	8.90E+	9.73E+	9.38E+	9.20E+	C010
FFA(18:1)	Lipids	8.20E+	1.31E+	8.83E+	2.39E+	C007
GcNeu	Organic	1.11E+	9.13E+	9.43E+	9.13E+	C034
Hydroxypyruvic acid	Organic	5.78E+	7.53E+	4.75E+	4.67E+	C001
Succinic acid	Organic	8.95E+	7.95E+	4.68E+	9.68E+	C000
12-KETE	Lipids	1.54E+	1.51E+	1.59E+	2.94E+	C148
1-Docosanoyl-sn-glycero-3-	GP	2.22E+	1.77E+	3.07E+	4.59E+	C042
5-Hydroxypseudobaptigenin	Heterocycl	1.87E+	2.00E+	1.92E+	1.93E+	C197
2-cis,6-cis-Farnesyl diphosphate	Organic	1.32E+	1.24E+	1.37E+	1.56E+	C197
dTDP-4-keto-2-deoxy-beta-L-	Nucleotide	7.03E+	7.28E+	7.32E+	1.48E+	C213
Premithramycin A3'	Benzene	1.11E+	9.16E+	9.35E+	8.61E+	C123
4-(beta-D-glucosyloxy)benzoic	Organic	5.56E+	5.71E+	5.31E+	4.83E+	C039
(3R)-3,8-dihydroxy-9-	Benzene	5.93E+	8.33E+	1.58E+	2.59E+	C124
Mannose 6-phosphate	Others	1.25E+	6.85E+	7.11E+	4.73E+	C002
Glucose	Others	1.91E+	1.83E+	1.74E+	1.85E+	C000
Dihydroechinofuran	Heterocycl	3.11E+	4.24E+	3.51E+	4.98E+	C181
LysoPC(18:3(6Z,9Z,12Z))	Lipids	6.54E+	1.97E+	8.04E+	5.07E+	C042
Docosanedioic acid	Lipids	2.15E+	2.23E+	2.86E+	1.58E+	C196
Aminodeoxyfutalosine	Nucleotide	8.40E+	7.53E+	8.24E+	9.84E+	C207
9,10-Dihydroxystearic acid	Lipids	1.44E+	1.83E+	3.38E+	6.13E+	C196
2-(alpha-D-mannosyl)-3-	Alcohol	3.29E+	1.92E+	3.03E+	2.66E+	C115
D-Gal alpha 1->6D-Gal alpha	Others	1.36E+	1.10E+	1.01E+	1.31E+	C054
lipoyl-AMP	Nucleotide	1.21E+	8.29E+	1.38E+	1.63E+	C162
4-Hydroxyphenylacetylglutamate	Amino	1.37E+	1.04E+	1.16E+	1.49E+	C055
(1E,3Z)-1-(3,4-dihydroxyphenyl)-	Phenolic	5.50E+	5.76E+	5.46E+	7.44E+	C177
Strigolactone ABC-rings	Others	2.34E+	2.99E+	2.96E+	2.02E+	C180
Melibiitol	Others	1.20E+	1.08E+	1.15E+	8.78E+	C053
S-Glutathionyl-L-cysteine	Amino	1.06E+	1.08E+	1.53E+	1.42E+	C055
2-Hydroxy-4-oxobutane-1,2,4-	Organic	3.47E+	3.33E+	3.55E+	3.06E+	C041
1-hexadecanoyl-2-(9Z-	GP	1.45E+	2.90E+	4.98E+	6.18E+	C001
1-Guanidino-1-deoxy-scyllo-	Organic	6.46E+	7.83E+	6.74E+	6.62E+	C012
Hydrogenobyrinate diamide	Organic	2.04E+	2.34E+	1.47E+	1.75E+	C065
Deacetylipecoside	Alcohol	2.09E+	2.50E+	1.69E+	2.28E+	C073
cis-Dihomoaconitic acid	Organic	4.14E+	4.20E+	4.17E+	4.05E+	C205
6'''-Deamino-6'''-oxoparomomycin	Heterocycl	6.96E+	1.31E+	7.09E+	5.81E+	C212
4,4'-Diapolycopenedial	Others	3.73E+	3.02E+	3.40E+	7.82E+	C197
Curcumin diglucoside	Steroids	1.69E+	1.45E+	1.70E+	1.49E+	C177

Data are the mean of three biological replicates.

Table. S2 Differential expression of key metabolites in the S. *xylocarpa* seeds under different storage condition.

Metabolite name	H-St vs L-Lt		H-Lt vs L-Lt		H-Lt vs L-Lt	
	Log2FC	P-	Log2FC	P-	Log2FC	P-
Vibriobactin	1.50	2.86E-	1.09	2.06E-	3.53E-	0.99
Sirohydrochlorin	1.18	6.73E-	1.07	2.71E-	1.78E-	0.95
1-Deoxyvaleric-acid	0.91	2.87E-	0.76	5.77E-	1.01E-	0.91
13-Hpode	-3.86	2.65E-	-2.76	1.12E-	6.95E-	-2.52
2-(3,7-Dimethyl-2,6-octadienyl)-3-	-4.40	4.53E-	-2.19	1.31E-	5.63E-	-1.53
dTDP-4-keto-2-deoxy-beta-L-xylose	4.40	7.52E-	1.03	1.89E-	1.78E-	1.03
16-Hydroxyhexadecanoic acid	-1.40	3.47E-	-1.64	1.04E-	1.79E-	-0.78
D-Glucuronic Acid	-1.64	1.10E-	-1.22	3.72E-	4.09E-	-0.79
L-Allothreonine	-1.19	4.45E-	-1.45	5.75E-	1.72E-	-0.76
9,10-Dihydroxystearic acid	-1.23	9.27E-	-1.57	1.99E-	4.76E-	-2.43
Glutathione Reducedform	0.74	2.72E-	1.13	7.32E-	1.04E-	4.21
Alangimarckine	0.66	2.72E-	1.60	6.35E-	7.79E-	0.95
Tetrahydromethanopterin	0.65	1.71E-	-1.79	3.43E-	2.68E-	3.20
Colchicine	-0.62	5.93E-	0.72	1.94E-	3.26E-	-4.60
Premithramycin A3'	-7.01	2.03E-	-6.73	1.37E-	2.81E-	-6.77
2-dehydro-D-gluconic acid	-9.15	2.51E-	-5.84	2.62E-	7.31E-	-3.90
N-Acetyl-L-aspartic acid	-0.89	1.72E-	-1.53	2.45E-	2.45E-	-1.53
Adenosine	-0.98	2.36E-	-1.17	4.84E-	2.29E-	-1.69
Premithramycin A2'	-1.09	1.27E-	-0.97	8.35E-	8.73E-	-0.60
L-cystathionine	0.79	3.26E-	1.95	9.74E-	2.96E-	2.10
Ononin	0.78	7.14E-	0.84	2.46E-	1.33E-	1.00
1-Palmitoyl-2-linoleoyl-sn-glycero-3-	-1.23	2.49E-	-1.79	4.44E-	5.79E-	-1.20
(±)12-HETE	-0.89	4.91E-	-0.83	4.67E-	4.67E-	-2.29
Vitexin 2''-O-rhamnoside	1.40	1.19E-	-1.29	3.70E-	1.11E-	-2.63
20-Hydroxyeicosatetraenoic acid	-1.75	1.32E-	-1.36	9.34E-	9.95E-	-2.04
alpha-Dimorphecolic acid	0.77	1.54E-	-2.11	2.67E-	1.69E-	-2.99
FFA(18:1)	-1.78	3.13E-	-2.46	1.77E-	3.69E-	-1.88
Glucaric acid	-2.06	4.79E-	-2.06	3.45E-	9.58E-	-2.13
Eicosadienoic acid	-2.18	2.01E-	-1.42	7.27E-	8.81E-	-1.83
1-Methyl-L-histidine	1.44	1.63E-	1.47	1.94E-	9.58E-	0.86
5-Methoxytryptamine	-3.06	1.36E-	-1.86	2.20E-	2.27E-	-1.85
Gluconic acid	-3.44	1.14E-	-2.57	4.10E-	9.10E-	-0.83
5'-Deoxy-5'-(Methylthio) Adenosine	-6.92	1.55E-	-5.27	7.09E-	7.09E-	-5.27
1-Docosanoyl-sn-glycero-3-	-2.27	8.65E-	-1.95	1.78E-	1.18E-	-2.75
Elloramycin	1.30	9.06E-	1.10	1.20E-	1.73E-	0.90
gamma-Linolenic Acid	-2.46	1.49E-	-2.29	2.46E-	3.71E-	-2.51
Adenylocuccinic Acid	1.34	8.68E-	0.87	1.92E-	5.61E-	0.94
4,4'-Diapolycopenedial	-2.25	2.23E-	-1.95	5.70E-	1.75E-	-2.17
2-Furancarboxaldehyde	-3.02	1.18E-	-1.21	3.93E-	1.63E-	-1.38
12-KETE	-2.39	6.87E-	-2.36	1.27E-	1.01E-	-2.44
1-Eicosanoyl-sn-glycero-3-	-3.14	3.63E-	-2.22	5.19E-	4.45E-	-2.12
1-Aminocyclopropanecarboxylic acid	-2.68	9.39E-	-2.74	7.35E-	1.63E-	-2.95
9(S),12(S),13(S)-TriHOME	-2.81	1.96E-	-1.61	1.21E-	8.61E-	-1.58
1-Heptadecanoyl-sn-glycero-3-	-2.18	8.19E-	-1.21	1.35E-	1.86E-	1.15

N1,N5,N10-tricaffeoyl spermidine	-4.97	1.86E-	-4.88	1.42E-	8.74E-	-3.75
Isopimaric acid	-3.18	7.86E-	-2.35	3.89E-	2.66E-	-2.16
LysoPC(20:1(11Z))	-0.68	6.68E-	-1.01	1.50E-	5.78E-	-0.85
FFA(18:2)	-0.69	5.27E-	-0.75	4.21E-	5.60E-	-2.15
